# Supplementary material for: Constructing the first comorbidity networks in companion dogs in the Dog Aging Project
Source: PLoS Comput Biol. 2025 Aug 14;21(8):e1012728. doi: 10.1371/journal.pcbi.1012728 (PMC12352663; doi:10.1371/journal.pcbi.1012728)
Supplement: Attachment — Submitted filename: response to reviewer comments.pdf [file pcbi.1012728.s009.pdf]

Response to Reviewers' Comments

We appreciate the detailed and constructive feedback from the reviewers. We have considered all the comments and made appropriate revisions to improve the manuscript. Below we provide point-by-point responses, where the reviewer's comments are in black and our responses are in blue.

Reviewer #1:

Thank you for this very interesting and well written manuscript. This paper describes in detail a methodology to identify comorbidities in canine patients through construction of comorbidity networks, using data obtained from the Dog Aging Project. This will potentially be of great help to clinicians in reaching diagnoses and anticipating concurrent conditions in their patients. It is also likely to be of interest to the public, especially pet owners who might recognise the comorbidities identified here in their own animals. The methodology appears sound and replicable, although I write this as a clinician-epidemiologist, so would recommend the editor also takes advice on this from a colleague in a suitable mathematical field as necessary.

Thank you for your thoughtful and constructive feedback. We appreciate your positive assessment of the manuscript and of our findings. We address each specific comment in detail below.

I have some minor comments which I hope you will find helpful.

1. Title: This paper focuses strongly on methodology rather than clinical application, and much of the information of interest to clinicians is relegated to the supplementary material. I feel that the title does not make clear this primary focus on methodology in its current form.

   We agree that this paper's focus is on methodology. We have modified the title to be "Constructing the first comorbidity networks in companion dogs in the Dog Aging Project" to reflect this point.

2. Line 74: Please could you explain the reason for choosing a cutoff of 60 dogs?

   We used the one-in-ten rule of thumb for logistic regression analysis, which states that one predictive variable should be studied for every ten events. Since we have 6 covariates in our model, we use a cutoff of 60 dogs [1]. This rule helps minimize the risk of overfitting. We have added a citation supporting this as well

as a brief addition to the “Logistic regression modeling” section in the Methods section:

“To minimize the risk of overfitting, we applied the one-in-ten rule of thumb for logistic regression analysis, which suggests studying one predictive variable for every ten events. Thus, we only included health conditions that occurred in at least 60 dogs, given the six covariates in our model.”

3. Line 80: Use of lbs is not standard in many countries, and the Plos Comp Biol author guidelines advise use of SI units. Please could SI units be added in parentheses throughout where imperial units have been used?

   We have added SI units (kilograms) in parentheses throughout the manuscript wherever pounds were used. For example, in line 80, we have revised “median weight of 50.8 lbs” to “median weight of 50.8 lbs (23.0kg)”.

4. Fig. 1, 2, and 3: These figures are not interpretable from a clinical perspective without supplementary table S1b. I wonder if this table needs to be included in the main body of the paper rather than as a supplement.

   Thanks for your suggestion! Taking the advice given by all reviewers, we made substantial changes to these figures to improve their interpretability. Specifically,

   - We have added node labels using the condition names instead of codes. This makes it much easier to understand the comorbidities.
   - To prevent clutter, we aggregated certain conditions as a single node so that more important comorbidities may be visually inspected. We were able to do so because conditions within the same category tend to be connected with each other. Aggregation primarily affects the skin and infection/parasites categories that tend to have many edges within each category. Whenever possible, we also included a zoomed-in view of the aggregated nodes so that all conditions within the aggregated categories are shown.
   - We colored the nodes by condition categories, and colored the edges to distinguish those connecting conditions within the same category from those connecting conditions from different categories. This modified color coding improves the visual assessment of the overall network structure.
   - We also included on GitHub a shiny app where the user can inspect the network with labels interactively.

   We hope that these improvements address your concern regarding the interpretability of our results.

5. Line 194: Fig S4 shows 13 mature adult and 1 senior comorbidity pairs, not ten and two?

Thank you for catching this typo; we have corrected it.

6. Line 283: How is age calculated? Does this relate to the onset of the condition, or start of treatment, or another date?

   Age is defined as age at enrollment, which is calculated from the date of birth (DoB) if known. For owners who do not know their dog's actual DoB, survey logic is used to collect the information they do have (e.g., year ± month of birth or current estimated age) to generate an estimated DoB [2].

   In other words, the age variable we consider is not related to the onset of any conditions.

7. Line 283: How is weight calculated? How are multiple episodes of weight recording in an individual patient record taken into account?

   We have cross-sectional data, so weight is only recorded once. Weight is an owner-reported variable (owners are prompted to give their dog's weight to the nearest pound to the best of their knowledge).

8. Line 383: Should this reference Fig. S4?

   Thank you for catching this mistake; this sentence should reference Fig 2 (age-stratified networks) instead of Fig S2 (node degree distribution). We have edited line 383 to reflect this.

9. Line 452: There is no Fig 4?

   Apologies for the typo; this line should reference Fig 3 (time-directed network) instead of Fig 4 (non-existent). We have edited line 452 to reflect this.

10. Line 501: The GitHub link is not currently active.

    Apologies for the oversight. The GitHub link was not active because it was set to be private instead of public. We have made the repository public, so the link works now.

Reviewer #2:

Summary: This paper entitled 'The first comorbidity networks in companion dogs in the Dog Aging Project' was a very interesting read and I really enjoyed it. The authors use mathematical approaches to understand comorbidities in dogs, which have rarely been applied within veterinary epidemiology. In addition to using these novel approaches, the paper provides useful insights into dog ageing and health. The paper is nicely written

and structured, with excellent grammar and no typing errors. I have a few comments about the reporting clarity, which at times makes it a challenge to understand the methods and results. At times, it is not clear why some results have been reported in the main text of the manuscript and discussed, and not others, which seem equally important.

Thank you for your positive assessment of our manuscript and recognition of our application of mathematical methods to veterinary epidemiology. We appreciate your thoughtful comments regarding reporting clarity and result selection. We have carefully considered your feedback and made substantial revisions to improve the clarity of our methods and results sections. Our point-by-point responses to specific comments are below.

- I take grievance to the authors referring to the data collection used in this study as ‘technically cross-sectional’ but able to ‘capture lifetime prevalence’. Although the long-term plan is for a longitudinal cohort, the Dog Aging Study at this point of data collection is a retrospective cross-sectional study and the authors should not try to infer otherwise (the study is referred to as longitudinal several times), which is confusing to the reader and ignores recall bias (although recall bias is later discussed). This ignores the fact that this type of study design is prone to survival bias, which is a major limitation that has been well-documented across many epidemiological studies, but isn’t included as a limitation in the discussion.

  We appreciate your comments regarding the study design. We want to clarify that 'lifetime prevalence' is a specific epidemiological term referring to the proportion of individuals who have experienced a particular condition at any point in their lives up to the time of the survey, regardless of whether the individuals are currently experiencing the condition or whether or not they have completed their lifespan. This is distinct from point prevalence (current active conditions) or period prevalence (conditions within a specific timeframe). At DAP enrollment, participants report all medical conditions their dogs have ever experienced, which by definition constitutes lifetime prevalence data collection.

  However, we still agree that our initial description was misleading in other respects. We have made changes throughout the manuscript to ensure clarity about the data being collected at a single point in time while being careful not to overstate what can be inferred from said data. Specifically:

  1. We have consistently revised our description of the study design, removing references to it as "longitudinal" or "technically cross-sectional." We now uniformly describe it as a "retrospective cross-sectional study" throughout the manuscript.

2. We have explicitly addressed survivor bias as a major limitation in the Discussion section: “Furthermore, the cross-sectional design is subject to survivor bias: only dogs who have survived to the point of entry into the study are included. This could distort the observed comorbidity patterns, especially for conditions like cancer that are associated with increased mortality, as dogs that died from these conditions prior to recruitment are not represented in our sample. As DAP collects longitudinal data, these issues may be partially addressed, but they remain a limitation of the current analysis.”

- Page 2, lines 49-50 “Notably, dogs are a good model organism because they share the same environments and many of the same health conditions as humans, they have an almost equally sophisticated healthcare system, and show similar manifestations of aging as humans” - I would suggest that dogs are in fact, treated much differently than humans - the onus in humans is to keep them alive as long as possible, whereas in veterinary healthcare it is far more about economic cost to the owner and also the moral/ethical considerations to the animal.

  It is true that owners have a wide range of options about how aggressively to treat medical and surgical problems in their dogs. They certainly can choose to pursue extremely sophisticated therapy, and they regularly do. Dogs routinely undergo joint replacement and repair; radiation therapy and chemotherapy; long-term management of endocrinopathies, kidney failure, liver failure and heart failure; cataract surgery and more. There is no other species for which this diversity of sophisticated care is as widely applied.

Results

- Although I like Figures 1, 2 and 3, it is impossible to understand the results using those figures alone, without referring to the supplementary material. Because of the magnitude of the results, it seems that many findings are easy to ‘lose’ and the author’s seem to have picked out certain results but do not explain many counter-intuitive ones. I wonder if some of the main results could be brought forward into the main manuscript to improve the reader experience and overall trust in what the author’s have chosen to discuss.

  Thanks for your suggestion! Taking the advice given by all reviewers, we made substantial changes to these figures to improve their interpretability. Specifically,

- We have added node labels using the condition names instead of codes. This makes it much easier to understand the comorbidities.
- To prevent clutter, we aggregated certain conditions as a single node so that more important comorbidities may be visually inspected. We were able to do so because conditions within the same category tend to be connected with each other. Aggregation primarily affects the skin and infection/parasites categories that tend to have many edges within each category. Whenever possible, we also included a zoomed-in view of the aggregated nodes so that all conditions within the aggregated categories are shown.
- We colored the nodes by condition categories, and colored the edges to distinguish those connecting conditions within the same category from those connecting conditions from different categories. This modified color coding improves the visual assessment of the overall network structure.
- We also included on GitHub a shiny app where the user can inspect the network with labels interactively.

We hope that these improvements address your concern regarding the interpretability of our results.

- Page 4 lines 83-84 "Additionally, most dogs have three or fewer health conditions, with just 37% of dogs having four or more health conditions" this seems like quite a large proportion to be phrased as 'just', perhaps rephrase? Additionally, these dogs technically have multimorbidity not comorbidity!

  Thank you for highlighting the tone and terminology issues in this sentence. We agree that our original phrasing ("just 37%") minimized a substantial proportion of dogs with multiple health conditions. We have made edits to remove this minimizing language and use the term "multimorbidity" to better describe dogs with multiple concurrent conditions.

  This sentence now reads: "Additionally, 28.4% of dogs have two or fewer conditions, while a substantial 71.6% exhibit multimorbidity with three or more concurrent health conditions."

- Page 4 Table 1 - please report percentages to 2 decimal places

  Thanks for your comment. Taking the advice given by Reviewer 3, we have deleted Table 1 and instead reported the information in text only. We have implemented your advice in the text as follows:

  "Sex and reproductive status were relatively balanced, with spayed females comprising 12,457 (46.81%) of the sample, and neutered males accounting for

12,196 (45.83%). Intact females (687, 2.58%) and intact males (1,274, 4.79%) were less common, with only 7.37% of dogs in the cohort being reproductively intact overall. The breed distribution was approximately equal, with 13,407 (50.38%) mixed breed dogs and 13,207 (49.62%) purebred dogs. The median age of dogs in the study was 7.75 (interquartile range [IQR]: 4.08-11.00 years), and the median weight was 50.90 lbs (IQR: 25.00-69.0 lbs [11.34-31.30 kg]).”

- Page 5 Table S2 - Cancers seem to have all important correlations with age and weight, with extremely low P-values reported across them all. This result seems to stand out to me, so why is it not reported?

  Thank you for noticing these important associations. Per your comment, we have added the following text in the Results section:

  “All cancer types showed significant positive associations for age ($P < 0.001$) and weight ($P < 0.05$).”

  Additionally, in the Discussion section, we included the following:

  “Our results reaffirm that age is the primary risk factor for many health conditions, while body size and breed background modulate disease risk in condition-specific manners. In our models, 80% of the coefficients in which age is significant are positive, confirming that morbidity rises steeply with older age. Other large epidemiological studies support this pattern [38,39]. Weight is significant in about 70% of the conditions, with 58% positive associations. Separate investigation using DAP data agreed that larger dogs had higher lifetime prevalence of cancer and orthopedic disorders but lower odds of ocular, cardiac, hepatic and respiratory disease [40]. For cancer specifically, heavier dogs were diagnosed several years earlier than lighter dogs [38]. These external findings corroborate our weight coefficients and clarify that “size risk” is condition-specific rather than universally detrimental.”

- I understand that not every result can be reported in the text here due to the magnitude of the results, but I would recommend that as a minimum, where results are reported in the text, that this is then discussed further in the discussion. Otherwise, it is not clear why these results were reported and not others. For example:
  Page 6 lines 107-109 “Purebred dogs showed significantly higher rates of ear infection, cataracts, and intervertebral disk disease.” This is not mentioned in the discussion - can we link to other research that shows similar findings?

  Thanks for your suggestion. Per your comment, we made substantial changes to the results and discussion section to ensure all reported results are discussed.

Specifically for the association between breed background and health conditions, we included the following in the Discussion section:

“The effects of breed background are highly condition-specific and not uniformly disadvantageous to purebreds. Consistent with our findings, a study based on the UK VetCompass cohort showed that ear infections were more common in purebreds [39]. A US study based on electronic health records of 27k dogs found purebreds over-represented in cataracts and IVDD, but no difference in hip dysplasia, patellar luxation, hypoadrenocorticism, hyperadrenocorticism, and mast cell tumors, while mixed-breed dogs had excess cruciate-ligament rupture and being hit by a car [41], which are aligned with our discoveries. On the other hand, their results on elbow dysplasia (bone/orthopedic) and hypothyroidism (endocrine), both over-represented in purebreds, are distinct from ours. This could be due to our use of a multivariate logistic regression model, where the effect of breed background is after adjusting for other covariates. ”

- Page 6 lines 108-109 “In comparison, mixed-breed dogs had significantly higher rates of cruciate ligament rupture, being hit by a car, pruritis, and hookworms.” - There is no further discussion of these results. Could this be that owners who own mixed breed dogs are less likely to worm them regularly or allow their dogs to roam freely more often? This is where owner-demographic data would have been a nice addition to this study, but I am not sure if this was possible.

Regardless of purebred vs mixed breed status, dogs are increasingly viewed as family members, and there is no evidence that owners of mixed breed dogs value them less, or practice a lower standard of husbandry, than owners of purebred dogs [3,4]. Based on the owner demographic data we have, we found that while mixed-breed dog owners were more likely to fall into lower income brackets, they actually reported higher rates of flea and tick prevention and similar rates of heartworm prevention compared to purebred owners, suggesting that income differences do not necessarily predict differences in preventative care. Additionally, dogs that are acquired as adults (e.g., from shelters) may have experienced these illnesses or injuries prior to acquisition by their current owners, and these dogs may be more likely to be mixed breed dogs.

In the revised manuscript, we added the following to the Discussion section:

“We found that mixed-breed dogs had higher rates of infectious and parasitic diseases. This pattern does not appear to stem from poorer husbandry: mixed-breed owners actually reported flea- and tick-control use more often than purebred owners and had comparable rates of heartworm prevention. Although mixed-breed owners were more likely to be in lower-income brackets, income alone therefore does not explain the difference. A plausible alternative is

acquisition history: mixed-breed dogs are more often adopted as adults—particularly from shelters—where they may have contracted infections or parasites before joining their current households."

- Page 10 lines 182-183 "This likely reflects the relatively low frequency of multiple health conditions in young dogs rather than simply a statistical power limitation" - this is a fairly big claim to make, did you do the power limitation calculation?

  We agree that this is an important distinction to clarify. We asserted that the lack of significant comorbidities in the puppy stratum reflects biological reality rather than just a statistical limitation based on an examination of our dataset's characteristics. The puppy cohort is small (n=476, 2% of total). We also observed that puppies only had 3 conditions that met the inclusion threshold (minimum 60 dogs per condition) and that 28% of the puppy cohort had comorbidities, compared with 77% of young adults, 90% of mature adults, and 95% of seniors. This aligns with clinical expectations that younger dogs generally experience fewer concurrent health conditions. We've added the following clarification in the manuscript to address this point:

  "Notably, no significant comorbidity patterns were detected for the puppy age group, which comprised less than 2% of the cohort. Puppies had only three conditions meeting the inclusion threshold and a much lower proportion of dogs with two concurrent conditions (28%) compared to other age groups."

- Page 10 lines 194-195 "Ten of these pairs came from the mature adult group and two from the senior group (Fig. S4).". Some of these are intuitive e.g lameness & osteoarthritis, but several are not - ticks and allergies, cataracts and deafness, conjunctivitis and giardia. There is no elaboration/discussion about these counter-intuitive associations and it does make me worry that some of the results feel 'cherry-picked'.

  In the revised manuscript, after updating the health conditions with additional preprocessing (by collapsing conditions that are listed twice under different categories), 14 of these pairs came from the mature adult group and 1 from the senior group. We included a detailed discussion on these results in the revised manuscript.

  "When comparing the unstratified network to the age-stratified versions, the unstratified network revealed numerous associations that were absent in the stratified analyses—likely due to reduced statistical power from smaller sample sizes. Conversely, the age-stratified networks uncovered several associations that did not appear in the overall network, highlighting patterns that may be specific to particular life stages. The ticks – gastrointestinal food/medicine

allergies pair is unique to the senior network. Neither condition is strongly associated with age, yet their comorbidity is only represented in the senior age group. The association between tick bites and alpha-gal syndrome (AGS) is well-documented in humans: the lone star tick is the primary source in transmitting the alpha-gal sugar that triggers AGS—an allergy to red meats and other mammalian products [PMID: 32928302]. In dogs, tick bites can also induce alpha-gal antibodies, but no clinical signs of food allergy were observed [PMCID: PMC6789585]. Further investigation is warranted to understand this comorbidity pair. The conjunctivitis – giardia pair unique to the mature adult network is unexpected. Given that both conditions involve mucosal inflammation, it could be that one or more genetic factor(s) make dogs susceptible to both conditions. Similarly, there may be a genetic component underlying the association between cataracts and deafness. For example, breeds carrying the merle allele (Australian Shepherd, Collie, Shetland Sheepdog, Great Dane, Dachshund, Catahoula Leopard Dog, etc.) have strict breeding recommendations that avoid mating two merles, preventing the high-risk M/M genotype that is associated with sensorineural deafness and ocular anomalies. Most of the common comorbidities identified by all networks regardless of stratification involve skin and/or gastrointestinal conditions that tend to have weaker—or even negative—associations with age than do cancer, endocrine, cardiac or brain/neurologic conditions. This suggests that while the unstratified network is valuable for broad discovery, combining it with stratification can provide further insights when focusing on specific conditions or specific groups of health conditions."

- Page 12 lines 226- 227 "we examined temporal relationships between conditions by computing the probability of one condition preceding another within a time window of 12 months" - in several places throughout the paper this time window of 12 months is referred to and even in the discussion there is mention of trying other time windows, but there is no justification of why the authors chose this time window of 12 months. Can you clarify? Were other time windows tested?

  Thank you for raising this important point about our choice of time window for our temporal analysis. We primarily based our choice of window size based on the advice given in the paper that we based our analysis method on [5]. To address your point, we modified the Results section to further explain our choice of window size:

  "Shorter window spans are more adept at capturing comorbidities that occur sequentially [18], but could result in power loss in comorbidity discovery since shorter window sizes encompass fewer dogs. Additionally, in shorter time windows, condition development, appearance of relevant signs and symptoms,

provider diagnosis, and the recording of said diagnosis may not be accurately captured. The ideal window size should be guided by clinical knowledge and research objectives. In our case, we chose a 12-month window to balance capturing meaningful temporal associations while minimizing potential confounding by intervening events or treatments."

Additionally, we tested a variety of alternative window sizes (1, 3, 6, 18, 24 months). We found that the resulting networks were largely consistent. This is illustrated in the heatmap below, which demonstrates the overlap in significant directed edges identified using different time windows.

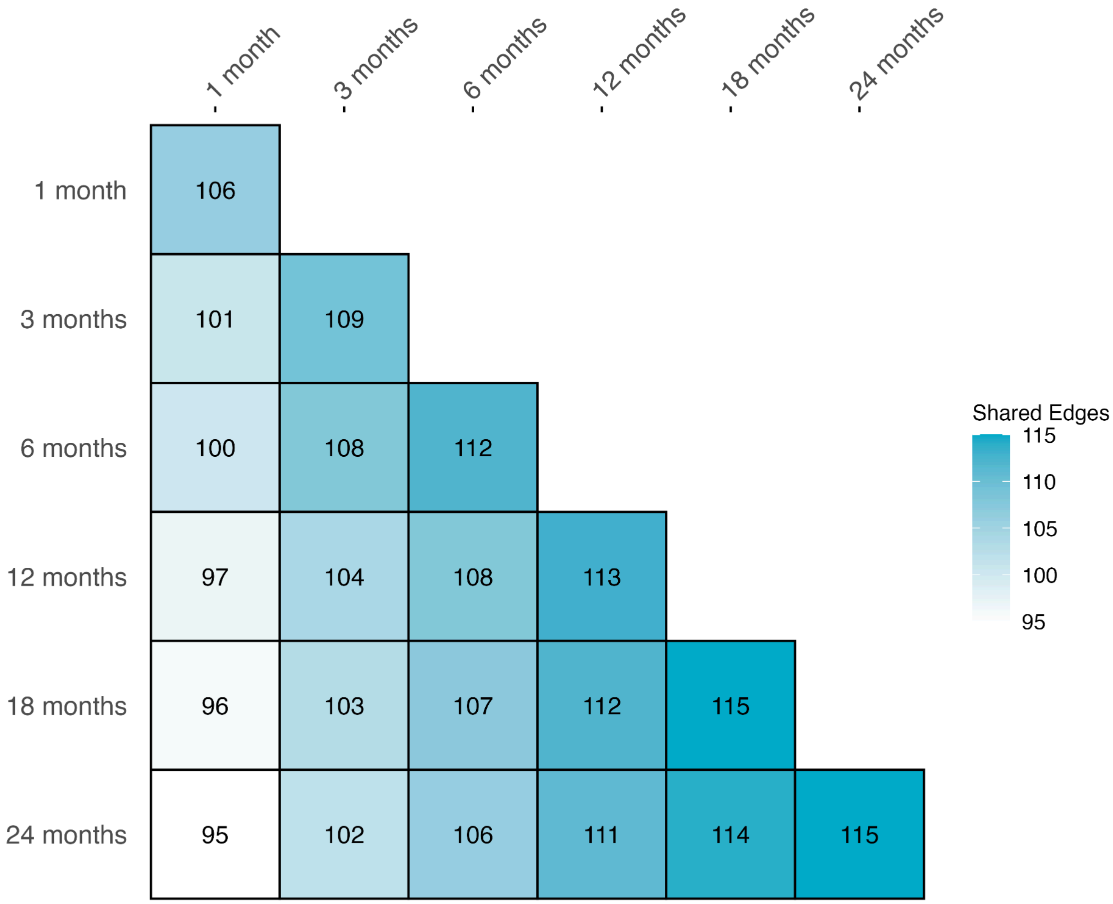

- Fig S1 is very useful for understanding the results and I would consider including it in the main manuscript. However, why is "chocolate" one of the most frequent diseases? This needs some explanation and perhaps adding the disease category it was assigned to (toxic consumption) would be useful (in addition to the other comorbidities).

Thank you for your suggestion. We've moved this figure to the main text as Figure 1. Additionally, we have clarified the axis label from "chocolate" to

“chocolate toxin consumption” to address the confusion. Table S1b also details the disease category that each condition belongs to, which can help clean up similar points of potential confusion. Your comment also reminded us that “condition” is more appropriate terminology than “disease” in many instances, as not all issues we analyze (such as toxin consumption) are diseases. Thus, we have also adjusted our word choice in the figure labels to reflect this distinction.

Methods

- Include a brief description of how you completed the analysis - in R software? What version? What packages were used? Where is the code available? What are your reporting statistics?

  All statistical analyses were performed using R version 4.5.0 (2025-04-11) in RStudio. We used the tidyverse package for data manipulation and visualization, and igraph for network visualization. Additional specialized packages included fastDummies for creating dummy variables, furrr for parallel processing, and poweRlaw for degree distribution analysis. Bonferroni correction was applied for multiple testing throughout our analyses.

  The complete code for reproducing all analyses and visualizations is available in our GitHub repository (https://github.com/aeyfang/pbc-dap-network/), which includes detailed documentation of the data processing workflow, statistical methods, and visualization techniques. We have amended the code availability section of the manuscript, essentially stating the above.

- Why were confidence intervals not reported? This would be useful for the reader.

  We did not include confidence intervals for the comorbidity results because the Poisson binomial test lacks a natural parameter for which confidence intervals can be constructed. This is similar to the Chi-squared test of independence. In 2×2 contingency tables, one can report confidence intervals for association measures such as the odds ratio, risk ratio, or phi-correlation. However, these measures do not generalize easily to settings where covariates influence individual disease risks. Therefore, we reported only the Bonferroni-adjusted p-values for each comorbid pair.

  For each network, we also provided a supplementary table reporting the test statistic along with the mean and variance of the corresponding null distribution

for each edge. We hope the extra information in these tables is helpful for the readers.

- Data cleaning is a prominent issue in questionnaire data - can you summarise how data were cleaned and processed?

  While survey logic means that not all respondents see all questions, our surveys do require a forced-choice response for every item that a respondent is presented with, and nearly all items allow for only a single response selection. This limits the need to clean the data. For the covariates sex, weight, and breed background, we used raw participant responses. The age covariate is defined as the difference between estimated date of birth (DOB) and HLES completion date. The DOB was estimated from a combination of owner-provided birth year and month, birth year, or age. In cases where erroneous birth year (potentially month) was provided, the record was updated based on other available data or by contacting the dog owner directly.

  In the previous submission, we did not perform any processing of the health status information. However, during revision we noticed that several conditions were intentionally offered twice under different disease categories/names because we were not sure where owners would look for it. Instead of treating them as distinct conditions, we collapsed them so as to learn more meaningful comorbidities. Specifically, laryngeal paralysis was included in both Neurologic and Respiratory, umbilical hernia was included in both Skin and Gastrointestinal, and intervertebral disc disease (IVDD) was included in both Neurologic and Orthopedic. In addition, degenerative joint disease and osteoarthritis are both offered, although they are used interchangeably in practice. In our analysis, we collapsed each pair of diseases so that laryngeal paralysis belongs to the Neurologic category, umbilical hernia belongs to Skin, IVDD belongs to Neurologic, and osteoarthritis belongs to Bone/Orthopedic. In addition, fractured bone was offered in five categories (long bone in limb, other bone in limb, spine, ribs, flat bone or head or face), which were collapsed into a single category. The cleaning on the health status explains the change in the number of dogs (n=26,614) and the number of conditions (n=160) included in the revised manuscript.

- Page 14/15 - lines 268-270 “As such, while the data we analyze are technically cross-sectional in nature because they were reported at a single point in time, the survey items are constructed to capture lifetime prevalence as defined above.” They are not ‘technically’ cross-sectional - they are cross-sectional. It is a

retrospective cross-sectional study. Please see my other comments about this and survivor bias.

We have modified our language to refer to our study as cross-sectional and discussed the limitation on survivor bias by adding the following text in the Discussion section:

“Furthermore, the cross-sectional design is subject to survivor bias: only dogs who have survived to the point of entry into the study are included. This could distort the observed comorbidity patterns, especially for conditions like cancer that are associated with increased mortality, as dogs that died from these conditions prior to recruitment are not represented in our sample. As DAP collects longitudinal data, these issues may be partially addressed, but they remain a limitation of the current analysis.”

- Page 15, lines 272-273 “Of note, the following information from this dataset was used: (1) age, sex, sterilization status, breed background, and weight of each dog”. Why were the following variables decided as confounders - age, sex, sterilization status, breed background, and weight of each dog, are there no other variables of relevance in the DAP?

  These variables were selected because they have been found to be associated with a dog’s risk of developing certain diseases [6–8]. DAP has also collected data on dogs’ diet, physical activity, environment, and owner demographics which may also be relevant for disease risk [9–11]. Due to the large number of potential risk factors, we have chosen to focus primarily on biological determinants of health in the current manuscript. Environmental and social determinants of canine health are important directions that we plan to explore in the future.

  We have added this limitation in the Discussion section as well:

  “Additionally, we only considered biological factors (age, sex, sterilization status, breed background, and weight) in disease risk prediction. Environmental and social factors can also affect disease risk [61] and should be incorporated in future analyses.”

- Page 15, lines 285-286. “We used breed background as opposed to breeds as a covariate because there are a large number of breeds and many health conditions are very rare.” How sure are you on breed background - are dogs registered (e.g with the Kennel Club or other breed associations) or is this just

owner reported? Have they been genetically tested or is it not possible to actually ascertain whether the dogs are pure-bred or not?

Breed background is owner-reported. Owners are prompted to select their dog's breed from a drop-down menu in the survey. While some dogs in the cohort have been genetically tested (via collected cheek swabs), this represents only a subset of the total cohort. We cannot determine purebred status through genetic verification for all dogs in our study. To address your concern, we also added a brief statement in our discussion of limitations:

"Fourth, breed classification (purebred vs. mixed-breed) is also owner-reported. While this binary classification eases statistical analysis, we cannot verify the accuracy of breed designations without genetic testing, which is only available for a subset of dogs in our cohort."

- Page 16 lines 264-266 "Owners are presented with more than 300 different health conditions or clinical signs, with an option for "other, please describe" in each body system, and also provide the time of onset for each reported condition" Was any effort made to identify/classify the diseases in the other category? It is not clear how this data was used as it is not reported in the results?

Thank you for pointing this oversight out. DAP has an ongoing effort to identify/classify the free-text responses in the "other, please describe" category. While a comprehensive analysis is unavailable, we can share some preliminary insights based on ongoing work.

Approximately 10% of respondents utilized the "other, please describe" option in the Health Status section. Of those that have been classified so far (just over half), 19.5% were instances of people writing the names of diseases that had been offered in the forced-choice lists (whether or not they also chose the forced-choice response). An additional 18.5% used the free text to share information that was not a medical condition, or to give details about a condition that had been selected from the list. Thus, close to 40% of the free-text responses didn't offer any new medical information. We recognize the potential value of these remaining responses and are committed to fully categorizing and incorporating this data in future work.

We also included a brief discussion about the free-text responses in the Discussion section to address this concern:

"We did not analyze the free-text responses provided by participants in the "other, please describe" fields. These free-text fields were used by about 10%

respondents and may contain valuable information. Future work will involve the systematic categorization and analysis of these responses."

- Page 18 line 58. Fig S3 is very useful for understanding the results, I would consider including it in the main manuscript, with overlaid cutoffs of the age categories that you defined explicitly to show young, mid and senior dogs - see my other comment about age categories.

  Thanks for your suggestion. We have included Fig S3 in the main manuscript (see the new Figure 1). However, we decided not to overlay the cutoffs of the age categories due to the varying stratification criteria by weight and breed (see our response to your next comment below).

- Page 19 lines 376-377 " We stratified the DAP dogs into four life-stage cohorts (from youngest to oldest): puppy, young adult, mature adult, and senior. These life stages were constructed using DAP veterinary guidelines" - State explicitly what the cut-offs were for these age categories. This will make the results easier to compare to other findings and also to understand how these figures relate to the information in Figure S3.

  We have added a table (shown below and Table S6 in the supplement) that explicitly shows the age cutoffs used for each life stage, which vary by dog weight class. This table was first published in [2]. The table presents age ranges in years for each life stage across five weight classes. We've included both imperial and SI units.

	Toy/Small (<22lb or <10kg)	Medium (22-<44 lbs or 10-<20kg)	Standard (44-<66 lbs, 20-<30kg)	Large (66-<88 lbs, 30-<40kg)	Giant (>88lbs or >40kg)
Puppy	≤0.75	≤0.75	≤0.75	≤1	≤1.5
Young Adult	>0.75-3	>0.75-3	>0.75-3 yrs	>1-3	>1.5-3
Mature Adult	>3-12	>3-12	>3-11 yrs	>3-10.5	>3-9.5
Senior	>12	>12	>11 yrs	>10.5	>9.5

- Page lines 459 - 462 "at least at the time of enrollment, dogs in the Dog Aging Project might be healthier than average if owners are disinclined to sign up a very unhealthy dog for a study on the determinants of healthy aging, similar to the well-known "healthy volunteer" effect in human studies". I agree, but this should not be understated. In addition to healthy volunteer bias, survival bias is

completely ignored here and is perhaps the most important limitation of this study. To elaborate, this sample comprises ONLY dogs who have survived to the point of entry into the study, (and no dogs that are already dead are included). This is a major limitation of the study design as it can distort the true relationships between comorbidities. This is reflected in the fact that there are few ‘fatal’ diseases reported in this study. For example, what about the dogs who died of cancer? I would like to see at least a couple of sentences discussing survivor bias, as it has huge implications for your findings.

Thanks for your valuable feedback. We have explicitly discussed survivor bias and its implications on our findings in the Discussion section:

“Furthermore, the cross-sectional design is subject to survivor bias: only dogs who have survived to the point of entry into the study are included. This could distort the observed comorbidity patterns, especially for conditions like cancer that are associated with increased mortality, as dogs that died from these conditions prior to recruitment are not represented in our sample. As DAP collects longitudinal data, these issues may be partially addressed, but they remain a limitation of the current analysis.”

- Page 21 lines 420-422 - “ For instance, this time-directed network exhibits a cluster of parasite-parasite interactions. While interest in co- or multiparasitism has increased in recent years, more work is needed to understand the mechanisms of these interactions” - These results are highlighted several times in the paper but I don’t think it is one of the more interesting findings. It is not surprising that several parasites are occurring together if owners simply are not worming their dogs?

  Thank you for your feedback on the overemphasis of parasite interactions. We agree that this finding, while methodologically apparent in our network, is not one of the more interesting, surprising results. In fact, these associations show up in all of our networks and can be simply explained. As suggested, we have de-emphasized this aspect of our findings by removing the extended discussion of parasite-parasite interactions.

- Page 21 lines 451 and 452 - “This issue likely explains some counterintuitive results like tick-borne health conditions preceding ticks in the time-directed network” - although the results are available in the figure cited, you have not reported this in the results text. Due to issues with the interpretability of the figures in their standalone form (see my comment at the start of results), this was not obvious when reading the results.

Thanks for raising this concern. In the revised manuscript, we have made sure that all results are first reported before being discussed. In the revised time-directed network, there are three counter-intuitive directional pairings: pneumonia before bordetella/parainfluenza, congestive heart failure before valve disease, and blindness before cataracts. We added the following text in the Discussion section:

"On the other hand, the time-directed network also reveals several counter-intuitive pairings: congestive heart failure → valve disease, pneumonia → bordetella/parainfluenza, and blindness → cataracts. One possible explanation for all three cases is reporting lag: owners may be informed of one condition and learn the specific cause later, reversing the apparent time-stamp. In the case of pneumonia → bordetella/parainfluenza, some dogs receive the bordetella/parainfluenza vaccine during hospital recovery, creating a false post-pneumonia "exposure" entry. "

The pair you mentioned: tick-borne health conditions preceding ticks is not in the revised network. After further checking, we realize that it may have been included due to a reporting error.

- Page 23, lines 462-463. "the owners in our study cohort are predominantly white, highly educated, and wealthier than the US population" It would be interesting to investigate the impact of this in future work.

  We agree! In this paper, we focused on more "biological" determinants of health, but socioeconomic factors are also very important. Since we claim earlier in the paper that dogs are good model organisms for humans partly because they share our environments, exploring owner demographics is a valuable direction for future work. We have looked at SES in other Dog Aging Project work, and in future could also consider the impact of SES on patterns of comorbidity [11].

- Finally, this paper focuses on comorbidity and pairs of diseases, but multimorbidity is of huge topical importance in the field of human research right now and it would be an interest area to develop and expand this work (e.g., using network analyses), so might be worth a mention!

  Thank you for helping us contextualize our manuscript within broader research interests. We added the following in our discussion of future work:

  "Furthermore, while our analysis focuses primarily on pairwise disease associations (comorbidities), many dogs in our cohort (71.6%) exhibit multimorbidity with three or more conditions. Future work could extend these network approaches to better understand complex interactions between multiple

diseases simultaneously. This direction would align with the growing interest in multimorbidity research in human medicine."

Reviewer #3:

Dear authors, the following are suggestions and comments:

- Table 1 is not necessary because it does not represent the sample. The data contained therein can be presented in text only.

  Thank you for this helpful suggestion. As recommended, we have removed Table 1 and integrated the demographic information into the text. The demographic characteristics of the study population are now presented as the following:

  "Sex and reproductive status were relatively balanced, with spayed females comprising 12,457 (46.81%) of the sample, and neutered males accounting for 12,196 (45.83%). Intact females (687, 2.58%) and intact males (1,274, 4.79%) were less common, with only 7.37% of dogs in the cohort being reproductively intact overall. The breed distribution was approximately equal, with 13,407 (50.38%) mixed breed dogs and 13,207 (49.62%) purebred dogs. The median age of dogs in the study was 7.75 (interquartile range [IQR]: 4.08-11.00 years), and the median weight was 50.90 lbs (IQR: 25.00-69.0 lbs [11.34-31.30 kg])."

- Unlike the data previously cited, the data in lines 90-112 could be better visualized and didactically presented by groups in a table, with real statistical confirmations.

  Thank you for this suggestion on our data presentation. We have created a table that presents the logistic regression model coefficients by disease category. The new table organizes health conditions into their respective categories (e.g., cardiac, respiratory, skin).

  For each health condition, we display the model coefficients for all demographic variables (age, weight, breed background, and sex/reproductive status). The statistical significance of each coefficient is clearly indicated using asterisks (* $p<0.05$, ** $p<0.01$, *** $p<0.001$), and we've implemented color-coding to enhance visual interpretation:

  - Dark green: Significant positive coefficients ($p<0.05$)
  - Light green: Non-significant positive coefficients
  - Light orange: Non-significant negative coefficients

- Red: Significant negative coefficients ($p<0.05$)

- I understand that Figure 1 is a complex figure, however it is an element that fails to present the data well because it is not self-explanatory. The use of acronyms and codes that are not described is a fundamental factor in this lack of clarity. I know that the authors may have a tendency to explain the figure as complex and with codes that are well known somewhere, however an incomprehensible graphic element is of no scientific use. I strongly recommend that the authors rethink Figure 1 so that it is didactic and actually demonstrates something, or remove it from the text (which is not the most recommended). Describe the legend in much more detail to facilitate this construction. Referring to Table S1 is also not a convenient idea.
- The same previous comment applies to Figures 2B, 2C and 3.

Thanks for your suggestion! Taking the advice given by all reviewers, we made substantial changes to these figures to improve their interpretability. Specifically,

- We have added node labels using the condition names instead of codes. This makes it much easier to understand the comorbidities.
- To prevent clutter, we aggregated certain conditions as a single node so that more important comorbidities may be visually inspected. We were able to do so because conditions within the same category tend to be connected with each other. Aggregation primarily affects the skin and infection/parasites categories that tend to have many edges within each category. Whenever possible, we also included a zoomed-in view of the aggregated nodes so that all conditions within the aggregated categories are shown.
- We colored the nodes by condition categories, and colored the edges to distinguish those connecting conditions within the same category from those connecting conditions from different categories. This modified color coding improves the visual assessment of the overall network structure.
- We also included on GitHub a shiny app where the user can inspect the network with labels interactively.

We hope that these improvements address your concern regarding the interpretability of our results.

- I really missed a robust conclusion about the data discussed. The data is robust, the analysis is also, and very rich in information, where the authors, in the end, did not even elaborate a conclusion with real applications on the data presented.

The aim of the current paper is to understand the relationship between aging and comorbidity. To this end, we discussed how comorbidity networks can reveal age-dependent variation. These results pave the foundation for further investigation into the causal relationship between aging and comorbidity.

In the revised manuscript, we added the following paragraph at the beginning of the Discussion section.

"Aging and comorbidities are linked in a self-reinforcing feedback loop [8]. Biological aging leads to increased risk of various diseases, which increases the risk of comorbidities. The presence of these comorbidities contributes to further physiological decline which accelerates biological aging. The dog is a great model to explore this relationship. Through the use of canine comorbidity networks, this study represents an important step towards understanding the relationship between aging and comorbidity. Our work also lays a critical foundation for incorporating future DAP genetic and other molecular data to elucidate the mechanisms underlying the association between aging and comorbidity. "

[1] Peduzzi P, Concato J, Kemper E, Holford TR, Feinstein AR. A simulation study of the number of events per variable in logistic regression analysis. J Clin Epidemiol 1996;49:1373–9. https://doi.org/10.1016/S0895-4356(96)00236-3.

[2] Pearson EB, Hoffman JM, Melvin RL, McNulty KE, Creevy KE, Ruple A. Analysis of 2,570 responses to Dog Aging Project End of Life Survey demonstrates that euthanasia is associated with cause of death but not age 2024. https://doi.org/10.2460/javma.23.07.0366.

[3] Turcsán B, Miklósi Á, Kubinyi E. Owner perceived differences between mixed-breed and purebred dogs. PLOS ONE 2017;12:e0172720. https://doi.org/10.1371/journal.pone.0172720.

[4] Kent M. 2024 AVMA Pet Ownership & Demographics Sourcebook. Schaumburg, IL: American Veterinary Medical Association; 2024.

[5] Lemmon G, Wesolowski S, Henrie A, Tristani-Firouzi M, Yandell M. A Poisson binomial-based statistical testing framework for comorbidity discovery across electronic health record datasets. Nat Comput Sci 2021;1:694–702. https://doi.org/10.1038/s43588-021-00141-9.

[6] Nam Y, White M, Karlsson EK, Creevy KE, Promislow DEL, McClelland RL. Dog size and patterns of disease history across the canine age spectrum: Results from the Dog Aging Project. PLOS ONE 2024;19:e0295840. https://doi.org/10.1371/journal.pone.0295840.

[7] Hoffman JM, Creevy KE, Promislow DEL. Reproductive capability is associated with lifespan and cause of death in companion dogs. PloS One 2013;8:e61082. https://doi.org/10.1371/journal.pone.0061082.

[8] Bellumori TP, Famula TR, Bannasch DL, Belanger JM, Oberbauer AM. Prevalence of inherited disorders among mixed-breed and purebred dogs: 27,254 cases (1995–2010) 2013. https://doi.org/10.2460/javma.242.11.1549.

[9] Bray EE, Zheng Z, Tolbert MK, McCoy BM, Akey JM, Benton B, et al. Once-daily feeding is

associated with better health in companion dogs: results from the Dog Aging Project. GeroScience 2022;44:1779–90. https://doi.org/10.1007/s11357-022-00575-7.
[10] Bray EE, Raichlen DA, Forsyth KK, Promislow DEL, Alexander GE, MacLean EL, et al. Associations between physical activity and cognitive dysfunction in older companion dogs: results from the Dog Aging Project. GeroScience 2023;45:645–61. https://doi.org/10.1007/s11357-022-00655-8.
[11] McCoy BM, Brassington L, Jin K, Dolby GA, Shrager S, Collins D, et al. Social determinants of health and disease in companion dogs: a cohort study from the Dog Aging Project. Evol Med Public Health 2023;11:187–201. https://doi.org/10.1093/emph/eoad011.
